# SGLT2 Inhibitors’ Effects on Biomarkers of Myocardial Injury: A Systematic Review

**DOI:** 10.34172/jcvtr.026.33445

**Published:** 2025-12-20

**Authors:** Elnaz Khani, Naser Aslanabadi, Haleh Rezaei, Hoda Afsharirad, Taher Entezari-Maleki, Sina Mashayekhi

**Affiliations:** ^1^Student Research Committee, Tabriz University of Medical Sciences, Tabriz, Iran; ^2^Cardiovascular Research Center, Tabriz University of Medical Sciences, Tabriz, Iran; ^3^Department of Clinical Pharmacy, Faculty of Pharmacy, Tabriz University of Medical Sciences, Tabriz, Iran

**Keywords:** Cardiovascular diseases, Myocardial injury, Sodium-glucose transporter 2 inhibitors

## Abstract

There is a lack of clinical data regarding sodium-glucose cotransporter-2 (SGLT2) inhibitors’ effects on myocardial injury, a common clinical condition that deteriorates patient prognosis. Hence, this systematic review aims to investigate the effects of SGLT2 inhibitors on myocardial injury, using the cardiac injury biomarkers, particularly high‐sensitivity cardiac troponin (hs-cTn). We performed a literature review on Embase, MEDLINE (PubMed), and the Cochrane Library, up to May 27, 2025. All potentially relevant randomized clinical trials (RCTs) assessing the effects of SGLT2 inhibitors on myocardial injury in individuals with diabetes or cardiovascular diseases were identified. The outcome measures were the most relevant cardiac injury biomarkers, including hs-cTnT and hs-cTnI, creatine kinase (CK)-MB, heart-type fatty acid-binding protein (H-FABP), myoglobin, lactate dehydrogenase (LDH), ischemia-modified albumin, and glycogen phosphorylase BB. In total, 1770 articles were identified through the literature search. After eliminating duplicates and screening articles, 10 studies involving 10440 participants were included. Of them, 6 studies evaluated hs-cTnT and 4 studies evaluated hs-cTnI. Among individuals with type 2 diabetes mellitus (T2DM), four of six trials showed significant attenuation in hs-cTn rise with SGLT2 inhibitors. In contrast, studies on HFrEF populations reported no significant change in hs-cTn levels. One study, including patients with acute myocardial infarction, also did not demonstrate a significant effect on hs-cTnI. The outcomes of this systematic review may suggest the potential effects of SGLT2 inhibitors in reducing myocardial injury by lowering hs-cTn levels in T2DM individuals. However, more studies are recommended to provide robust evidence.

## Introduction

 Myocardial injury is defined as the impaired cardiomyocyte membrane integrity leading to the release of various biologically active cytosolic and structural proteins into the extracellular space. Myocardial injury may be acute, as evidenced by dynamic changes in cardiac biomarkers, or chronic, in which biomarkers are persistently elevated. Both structural cardiac diseases, particularly heart failure (HF), and noncardiac conditions, such as diabetes mellitus, may contribute to chronic myocardial injury.^1,2^ Evidence has shown that myocardial injury in the absence of acute coronary syndrome is a common clinical condition that deteriorates patient prognosis.^3^

 Sodium-glucose cotransporter-2 (SGLT2) inhibitors are a class of anti-diabetic medications that have gained attention for their cardiovascular benefits.^4^ The potential mechanisms by which they exert cardioprotective effects include increasing circulating ketone body levels, mitigating oxidative stress and inflammation, osmotic diuresis, attenuating the renin-angiotensin-aldosterone system, and inhibiting the sodium-hydrogen exchanger (NHE) pump.^5,6^

 Clinical evidence regarding the effects of SGLT2 inhibitors on myocardial injury is still lacking and mostly limited to animal studies. In a rat model study, it was indicated that canagliflozin is associated with a significant reduction in infarct size through ameliorating myocardial ischemia/reperfusion injury.^7^ Another animal model study showed that empagliflozin protects the heart against coronary microvascular dysfunction and improves coronary flow reserve.^8^

 Although evidence regarding physiological processes that connect SGLT2 inhibitors to indicators of myocardial damage is still lacking, some hypotheses have been made. It is suggested that SGLT2 inhibitors directly inhibit the NHE1 isoform in the myocardium, the activity of which is markedly increased in patients with HF. Hence, NHE inhibition may result in decreased fibrosis, hypertrophy, systolic dysfunction, remodeling, and cardiac injury.^9^ SGLT2 inhibitors may also reduce cardiac wall stress, necrosis, fibrosis, and ventricular loading by decreasing preload and afterload.^9^ Such alterations may lead to a reduction in myocardial injury. Other mechanisms that link SGLT2 inhibitors to indicators of myocardial damage may be attributable to a decrease in inflammation, oxidative stress, and cellular apoptosis, which are key contributors to myocardial damage. They also improve oxygen supply to cardiomyocytes, impacting both functional and morphological aspects of the myocardium.^6^

 Several biomarkers have been shown to rise following myocardial injury.^10,11^ Given the lack of clinical data regarding the effects of SGLT2 inhibitors on myocardial injury, we performed this systematic review to explore their effects on the most relevant cardiac injury biomarkers. The primary outcomes were high‐sensitivity cardiac troponin (hs-cTn), creatine kinase (CK)-MB, heart-type fatty acid-binding protein (H-FABP), myoglobin, lactate dehydrogenase (LDH), ischemia-modified albumin, and glycogen phosphorylase BB.

## Materials and Methods

###  Study design

 The present study is designed according to the Preferred Reporting Items for Systematic Review and Meta-Analysis (PRISMA) reporting guidelines to assess the effects of SGLT2 inhibitors on cardiac injury biomarkers in patients with cardiovascular diseases or diabetes. The protocol of the systematic review was registered on PROSPERO (ID: CRD42024573051).

###  Selection criteria and search strategy

 In this study, all potentially relevant randomized clinical trials (RCTs) published up to May 27, 2025, were identified by searching PubMed (Medline), Embase, and Cochrane Library. Emtree and medical subject headings (MeSH) were used to select relevant keywords. The search strategy is accessible in supplementary Table S1.

 The reference lists of relevant studies were checked to avoid missing eligible articles. The Population, Intervention, Comparison, Outcome (PICO) framework was used to express eligibility criteria. All RCTs involving individuals with diabetes or cardiovascular diseases that assessed SGLT2 inhibitors’ effects on cardiac injury biomarkers, including hs-cTn, CK-MB, H-FABP, myoglobin, LDH, ischemia-modified albumin, and glycogen phosphorylase BB, were included. The type of SGLT2 inhibitors included in the search strategy were canagliflozin, atigliflozin, bexagliflozin, dapagliflozin, empagliflozin, enavogliflozin, ertugliflozin, ipragliflozin, licogliflozin, luseogliflozin, mizagliflozin, remogliflozin, sergliflozin, sotagliflozin, and tofogliflozin.

 Studies lacking a control group, review articles, letters to the editor, commentaries, case reports, animal studies, laboratory and in-silico studies, duplicate publications, and articles in languages other than English were excluded from the study.

 Two reviewers (EK and HA) independently screened the records for their potential *inclusion* against the *eligibility criteria* to extract data using a standardized checklist adapted from the Cochrane Collaboration’s data collection form. Any disagreements were discussed with the corresponding author, TE.

 Data extracted were 1) study general information, including first author’s name, year, country, design, and follow-up duration 2) individuals’ characteristics, including mean age, sex, and health condition 3) intervention, including dose, duration, and route of administration 4) The level of cardiac injury biomarkers as outcomes.

###  Risk of bias calculation

 The scale used to evaluate the risk of bias was the Revised Tool for Risk of Bias in Randomized Trials (RoB 2), which is designed to evaluate the risk of bias across five domains: (1) bias arising from the randomization process, (2) bias due to deviations from intended interventions, (3) bias due to missing outcome data, (4) bias in measurement of the outcome, and (5) bias in selection of the reported result. For the crossover trial included in our review, additional considerations for period and carryover effects were applied following Cochrane guidance.^12,13^ Two reviewers (E.K. and H.A.) independently assessed the risk of bias for each included study. Discrepancies in judgment were resolved through discussion and consensus, and, if necessary, consultation with a third reviewer (T.E.). This process was conducted manually, without the use of any automation or machine learning tools. We used the robvis tool to generate traffic-light plots and summary figures of the overall risk of bias judgments across studies.^14^

## Results

###  Study selection process

 Overall, 1770 articles were retrieved through database search and manual screening. After removing duplicate records and screening articles by titles or abstracts, 1672 studies were excluded. Of the remaining 98 studies, 88 were excluded after the full-text screening. Finally, 10 studies with 10440 participants were included in this systematic review.^15-24^ The selection process and reasons for exclusion are presented in a PRISMA flow diagram (Figure 1).

**Figure 1:**
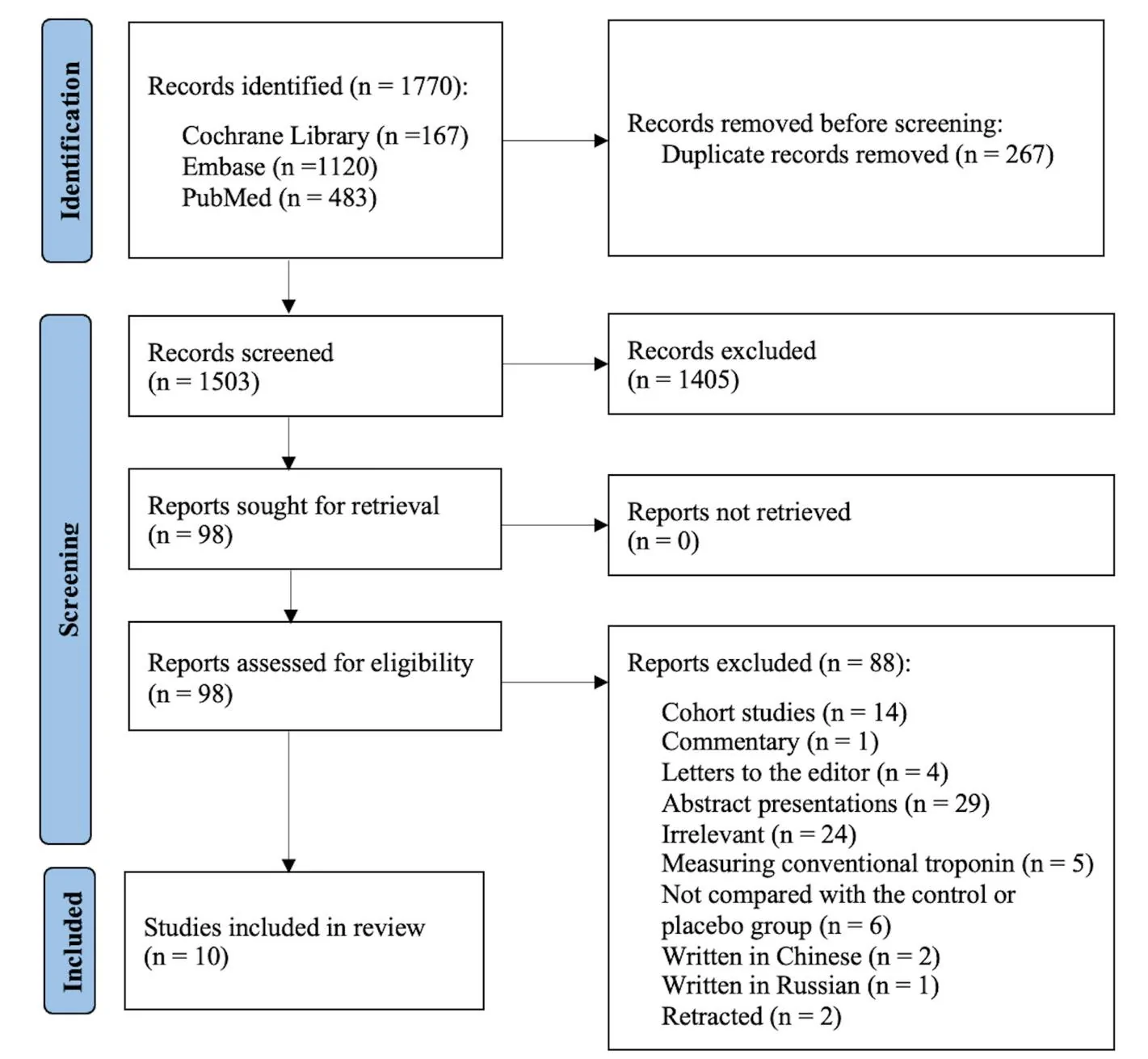
Preferred reporting items for systematic reviews and meta-analyses flow diagram of the literature search process

###  Study characteristics

 All included studies were parallel-group except for the study by Ilyas et al which was a crossover trial.^17^ Four studies were multinational, and the remaining studies were conducted in the United Kingdom, Australia, Denmark, Portugal, Iran, and Japan (one study for each country). Overall, 5945 patients received SGLT2 inhibitors, and 4476 patients received placebo in the parallel-group studies. All included studies evaluated SGLT2 inhibitors’ effects on hs-cTn levels as the marker of myocardial injury and were published from 2017 to 2024. The types of investigated SGLT2 inhibitors were empagliflozin (4 studies), dapagliflozin (3 studies), and canagliflozin (3 studies). The age of patients ranged from 60 to 73 years. The detailed characteristics of each study are shown in Table 1.

**Table 1:** Baseline characteristics of included RCTs

**First author, year**	**Country**	**Design**	**Population**	**Total sample size (intervention, control)**	**Age, year**	**Female sex, n (%)**	**Intervention**	**Compare**	**Follow-up**
Berg et al.^15^, 2022	Multinational	Parallel-group double-blind	HFrEF	3112 (1573, 1539)	67.2 ± 10.4	677 (21.8)	Dapagliflozin 10 mg daily	Placebo	12 months
Carberry et al.^16^, 2024	United Kingdom	Multicenter double-blind	Type 1 acute myocardial infarction	104 (51, 53)	63.0 ± 11.2	14 (13)	Empagliflozin 10 mg daily	Placebo	24 weeks
Ilyas et al.^17^, 2021	Australia	Crossover double-blind	T2DM and HFrEf	19	73 (63, 81)	5 (26)	Dapagliﬂozin 10 mg daily	Placebo	2 weeks
Januzzi et al.^18^, 2017	Multinational	Parallel-group double-blind	T2DM	666 (450, 216)	Intervention: 64.0 (6.3); Placebo: 63.2 (6.3)	Intervention: 202 (45); Placebo: 83 (38)	Canagliﬂozin 100 and 300 mg	Placebo	104 weeks
Januzzi et al.^19^, 2023	Multinational	Parallel-group double-blind	T2DM and CKD at a high risk of progression	2627 (1324, 1303)	Intervention: 63.2 ± 9.1; Placebo: 63.3 ± 9.2	Intervention: 451 (34.1); Placebo: 434 (33.3)	Canagliflozin 100 mg	Placebo	3 years
Omar et al.^20^, 2022	Denmark	Parallel-group double-blind	HFrEF	187 (94, 93)	64 ± 11	28 (15)	Empagliflozin 10 mg daily	Placebo	12 weeks
Reis et al.^21^, 2022	Portugal	Single-site open-label	Non-diabetic patients with HFrEF	40 (20, 20)	60.9 ± 13.0	7 (17.5)	Dapagliflozin 10 mg daily	Conventional treatment	6 months
Taheri et al.^22^, 2023	Iran	Multicenter double-blind	T2DM with coronary artery disease	77 (42, 35)	Intervention: 62.07 ± 7.34; Placebo: 60.58 ± 7.54	Intervention: 21 (50.0); Placebo: 22 (62.9)	Empagliflozin 10 mg daily	Placebo	26 weeks
Tanaka et al.^23^, 2021	Japan	Multicenter double-blind	T2DM with cardiovascular disease	105 (52, 53)	64.8 ± 10.4	33 (31.4)	Empagliflozin 10 mg daily	Placebo	24 weeks
Vaduganathan et al.^24^, 2022	Multinational	Multicenter double-blind	T2DM at high risk for cardiovascular events	3503 (2339, 1164)	63 ± 8	1171 (33.4)	Canagliﬂozin 100 or 300 mg	Placebo	6 years

Data are presented as n (%), mean ± SD, or median (IQR, 25th-75th percentile). CKD, chronic kidney disease; HFrEF, heart failure with reduced ejection fraction; IQR, interquartile range; SD, standard deviation; STEMI, ST-segment elevation myocardial infarction; T2DM, type 2 diabetes mellitus

###  Risk of bias results

 Six out of nine studies had an overall low risk of bias. One study had some concerns regarding the implemented method for the data analysis. Three studies had an overall high risk of bias due to problems with the randomization process, concerns arising from the implemented method for the analysis of data, and missing outcome data, or period and carryover effects in the crossover trial.^17,21,23^ Detailed quality assessment results are summarized in Figure 2.

**Figure 2:**
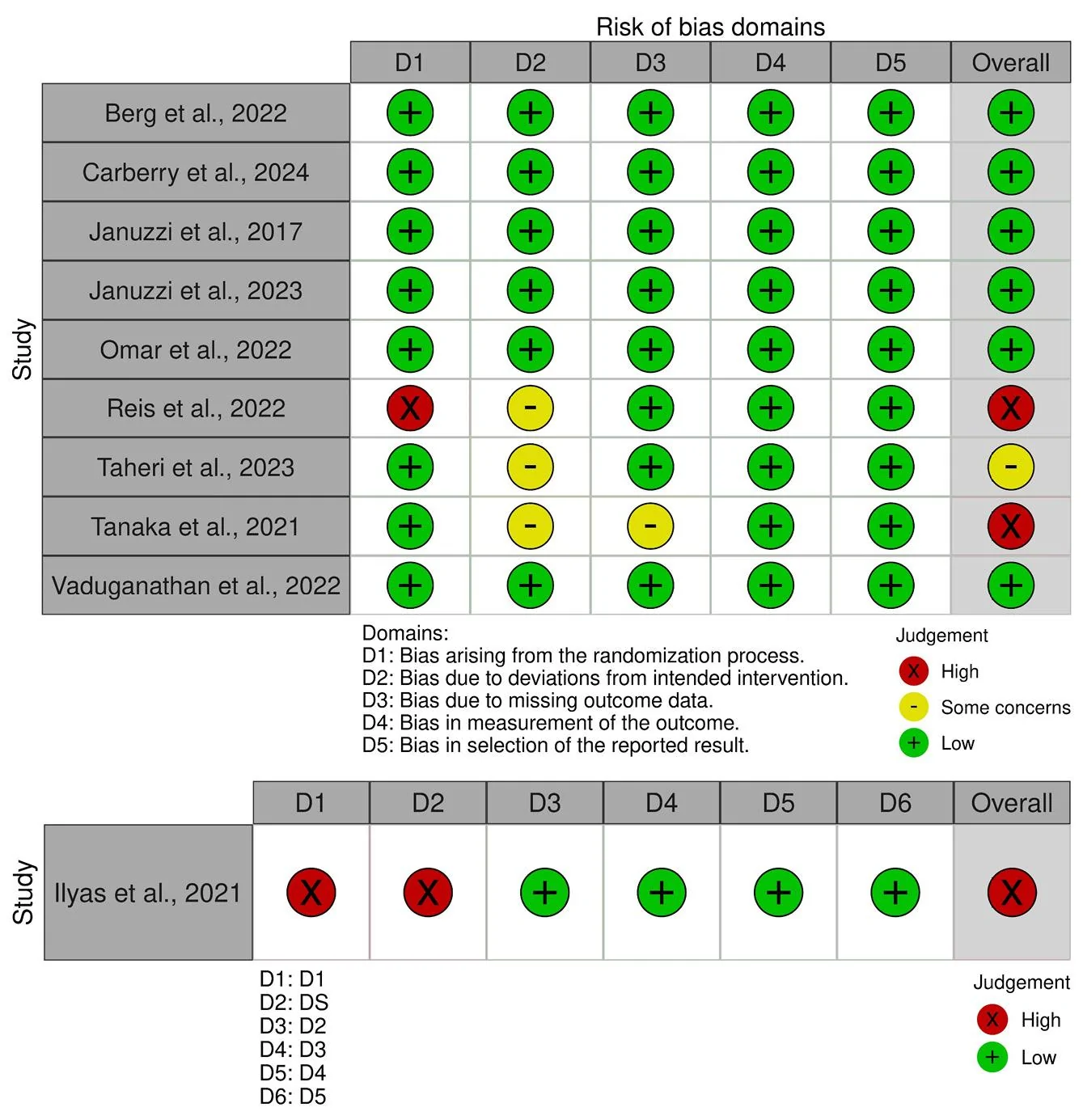
The results of risk of bias assessments by RoB 2 in the systematic review

###  Findings

 In a study by Berg et al., dapagliflozin attenuated the rise in hs-cTnT over 12 months in patients with left ventricular ejection fraction (LVEF) ≤ 40% and New York Heart Association class (NYHA) II to IV symptoms (n = 3112).^15^ However, the difference was not statistically significant compared to the placebo group (*P* = 0.076). The median (interquartile range [IQR]) baseline hs-cTnT concentration was 20.0 (13.7, 30.2) ng/L in the total population. For those with available hs-cTnT at both baseline and 12 months without a history of myocardial infarction (MI) or any episode of worsening HF within 30 days before the 12-month study visit (n = 2400), the baseline median hs-cTnT levels were changed from 18.8 to 19.5 ng/L and 19.6 to 19.2 ng/L at 12 months in the placebo and intervention groups, respectively. In a crossover trial of 19 patients with type 2 diabetes mellitus (T2DM) and heart failure with reduced ejection fraction (HFrEF), Ilyas et al showed that the median level of hs-cTnT was increased in dapagliflozin-treated patients compared to the placebo [25 (19, 37) vs. 28 (20, 42) ng/L, *P* < 0.01].^17^ In this trial, the baseline value of hs-cTnT was 24 (18, 39) ng/L. Another study by Omar et al revealed that administration of empagliflozin in 187 patients with HFrEF was not associated with a significant change in hs-cTnT levels compared to the placebo when adjusted for age, sex, and diabetes (ratio of change: 1.07; 95% confidence interval [CI], 0.98 to 1.19).^20^ In this study, the baseline median hs-cTnT levels of patients were 12.9 and 14.2 ng/L in the intervention and control groups, respectively. In a study by Reis et al., which included 40 non-diabetic patients with HFrEF, the mean hs-cTnI level was decreased in the dapagliflozin-treated patients from 9.2 to 8.4 ng/mL, while it increased from 21.9 to 27.7 ng/mL in the conventional treatment group at 6-month follow-up period.^21^ However, the difference was not significant between the two groups (*P* = 0.2).

 In a study including 666 older patients with T2DM, Januzzi et al showed that canagliflozin significantly attenuated the rise in hs-cTnI serum levels over 2 years, compared with placebo (*P* < 0.01).^18^ The median level of hs-cTnI at baseline was approximately 3.3 pg/ml in both groups. The between-group difference in median percent change at weeks 26, 52, and 104 were -8.3% (95% CI, -14.0% to -2.5%), -11.9% (95% CI, -18.0% to -5.6%), and -10.0% (95% CI, -17.3% to -2.6%), respectively.

 In another study by Januzzi et al on 2627 T2DM patients with chronic kidney disease (CKD), canagliflozin significantly reduced the rise in hs-cTnT, as evidenced by a geometric mean ratio of 0.96 (95% CI, 0.93 to 0.99; *P* = 0.002), expressing the adjusted relative difference in concentration at 1 year compared to baseline.^19^ The baseline median level of hs-cTnT was 19 ng/L in each group, which increased to 21 and 23 ng/L in the intervention and placebo groups at the end of 3 years, respectively.

 Tanaka et al. included 105 T2DM patients with cardiovascular disease and evaluated the effects of empagliflozin on hs-cTnI, stratified based on the baseline metformin use.^23^ No significant randomization-based group ratios of proportional changes in the geometric mean of hs-cTnI were observed from baseline to week 24 between the empagliflozin and placebo groups (metformin users: 0.93; 95% CI, 0.74 to 1.16, *P* = 0.49; nonusers: 0.96; 95% CI, 0.73 to 1.27, *P* = 0.78).

 In another study on 3503 individuals with T2DM at high risk for cardiovascular events, Vaduganathan et al showed that hs-cTnT increased in the placebo and canagliﬂozin groups up to 1 year with similar trajectories. However, canagliﬂozin signiﬁcantly lowered the rise in hs-cTnT levels at year 6 compared with placebo (geometric mean ratio, 93.8%; 95% CI, 88.6 to 99.3; *P* = 0.027).^24^

 According to Taheri et al study on 77 patients with T2DM with coronary artery disease, the mean level of hs-cTnI (ng/L) decreased from 22.85 to 9.77 in empagliflozin recipients and increased from 22.39 to 23.01 in the placebo group throughout the 26 weeks study period (mean difference: -13.24; 95% CI, -14.15 to -12.33; *P* < 0.001).^22^

 In the EMPRESS-MI randomized, double-blind, placebo-controlled trial, 105 patients with post-MI LVEF < 45% were randomized to receive empagliflozin 10 mg daily or placebo for 24 weeks. Both the empagliflozin and placebo groups exhibited a significant reduction in hs-TnI concentrations over the study period. However, there was no statistically significant between-group difference in hs-TnI change at 24 weeks (adjusted ratio of geometric means: 1.10, 95% CI 0.81–1.50; *P* = 0.54).^16^ The detailed outcomes and results can be found in Table 2.

**Table 2:** Outcomes of the included studies

**Study**	**Biomarker (unit)**	**Efficacy measure(s)**	***P*** **-value**
Berg et al.^15^, 2022	hs-cTnT (ng/L)	Relative least squares mean reduction from baseline to 12 months (95% CI): -3% (-6% to 0%)	0.076
Carberry et al.^16^, 2024	hs-cTnI (ng/L)	Between-group difference reported as ratios of adjusted geometric means (95% CI): 1.10 (0.81, 1.50)	0.54
Ilyas et al.^17^, 2021	hs-cTnT (ng/L)	Comparison of variation between groups (median [IQR]): increase in the intervention group from 24 (18, 39) to 28 (20, 42) vs. control group from 24 (18, 39) to 25 (19, 37)	< 0.01
Januzzi et al.^18^, 2017	hs-cTnT (pg/ml)	Difference in median percent change from baseline over 104 weeks (95% CI): -10.0% (-17.3% to -2.6%)	< 0.01
Januzzi et al.^19^, 2023	hs-cTnT (ng/L)	Treatment-related effect as geometric mean ratio expressing adjusted relative difference in concentration at 1 year vs. baseline (95% CI): 0.96 (0.93 to 0.99)	0.002
Omar et al.^20^, 2022	hs-TnT (ng/L)	Adjusted between-group treatment effect as a ratio of change (95% CI): 1.07 (0.98 to 1.19)	0.18
Reis et al.^21^, 2022	hs-cTnI (ng/mL)	Comparison of variation between groups (mean ± SD): decrease in the intervention group from 9.2 ± 6.7 to 8.4 ± 5.2 vs. increase in the control group from 21.9 ± 45.1 to 27.7 ± 43.4	0.2
Taheri et al.^22^, 2023	hs-cTnI (ng/L)	Mean difference of changes between groups (95% CI): -13.24 (-14.15 to -12.33)	< 0.001
Tanaka et al.^23^, 2021	hs-cTnI (pg/mL)	Proportional changes in the geometric mean from baseline to week 24 according to the baseline metformin use and randomization group (95% CI): metformin users: 0.93 (0.74 to 1.16); nonusers: 0.96 (0.73 to 1.27)	Metformin-users: 0.490 nonusers: 0.788
Vaduganathan et al.^24^, 2022	hs-cTnT (pg/mL)	Geometric mean ratio at year 6 compared with placebo (95% CI): 93.8% (88.6 to 99.3)	0.027

*P*-value inicates difference vs. placebo. AUC, area under the curve; CI, confidence interval, cTn, cardiac troponin; hs-cTn, high-sensitivity cardiac troponin; IQR, interquartile range; SE, standard error; SD, standard deviation

## Discussion

 To our knowledge, this is the first systematic review addressing the effects of SGLT2 inhibitors on cardiac injury biomarkers. All included studies focused on hs-cTn, the most sensitive and specific biomarker for detecting myocardial injury. The findings suggest that SGLT2 inhibitors may reduce hs-cTn levels in patients with T2DM, whereas the evidence is less consistent in HF patients.

 Since the exact mechanism by which SGLT2 inhibitors exert their cardioprotective effects is unknown, changes in biomarker levels may provide insights into mechanisms of beneﬁt. Previous research has predominantly assessed SGLT2 inhibitors’ effects on natriuretic peptides as indicators of cardiac dysfunction, but data on biomarkers specific to myocardial injury are limited.^25^ Among available cardiac injury biomarkers, hs-cTn appears to be the most sensitive and specific biomarker.^26,27^ hs-cTn not only shows overt myocardial necrosis but also can detect myocardial injury before experiencing symptoms and acute coronary syndrome.^28^ According to a meta-analysis of 28 studies including 154052 asymptomatic participants, even a minimal rise in hs-cTn levels was associated with cardiovascular mortality.^29^ Several biomarker-driven prognostic models also revealed that hs-cTn rise was an independent predictor of cardiovascular mortality and hospitalization in patients with HF and diabetes.^30,31^

 Several studies in this systematic review support the role of SGLT2 inhibitors in reducing hs-cTn levels in TD2M patients. For example, Januzzi et al demonstrated that canagliflozin attenuated hs-cTnI elevation over 104 weeks in adults with long-standing T2DM.^18^ A sensitivity analysis confirmed the findings despite missing data at later time points. However, this study excluded patients with estimated glomerular filtration rate (eGFR) < 50 ml/min/1.73 m^2^, limiting generalizability to those with impaired renal function. To address this issue, another study by Januzzi et al included T2DM patients with CKD, in which SGLT2 inhibitors could decrease hs-cTnT levels.^19^

 Conversely, Tanaka et al which also included individuals with T2DM and cardiovascular disease, found no significant difference in hs-cTnI levels between the empagliflozin and placebo groups.^23^ The study’s limited sample size and lack of adjustment for confounders may have undermined its power and internal validity.

 Four out of six studies that did not support the beneficial effects of SGLT2 inhibitors on hs-cTn levels included patients with HFrEF. For example, Ilyas et al found a significant increase in hs-cTnT following dapagliflozin administration.^17^ However, the study’s small sample size and bias arising from the period or carryover effects and the randomization process make interpreting the results difficult. Similarly, Reis et al study on non-diabetic individuals with HFrEF reported a non-significant reduction in hs-cTnI levels.^21^ However, issues related to randomization and missing outcome data may have limited its statistical power and reliability.

 A biomarker substudy of the DAPA-HF trial by Berg et al demonstrated a nonsignificant trend toward mitigation of the rise in hs-cTnT in HFrEF patients treated with dapagliflozin.^15^ Noteworthy, nearly all participants in this trial had a noticeable myocardial injury at the time of study admission, which may have limited the observable treatment effect. The authors mention that although the difference in hs-cTnT elevation between treatment groups was small, even minor changes in hs-cTnT may carry clinical significance. In this trial, patients with an eGFR < 30 ml/min/1.73 m^2^ were excluded. Hence, the cut-off points used in this study for hs-cTnT levels cannot be applied to patients with impaired renal function.

 The variability in findings observed across the included studies may suggest that the protective effects of SGLT2 inhibitors in reducing hs-cTn are more pronounced in individuals with metabolic risk factors, such as T2DM, compared to those with existing structural heart disease and cardiac remodeling, where myocardial injury may be more advanced and less modifiable by pharmacological interventions.

 For example, the EMPRESS-MI trial, which included patients with systolic dysfunction after acute MI, found no additional reduction in hs-TnI levels with empagliflozin compared to placebo over 24 weeks.^16^ These findings suggest that SGLT2 inhibitors may not substantially impact ongoing myocardial injury as reflected by hs-cTn levels, in the early recovery phase post-MI.

 The current systematic review is the first study that supports the beneficial effects of SGLT2 inhibitors in reducing hs-cTn levels as the preferred biomarker of cardiac injury. The results of this study can assist researchers in designing future studies by addressing the limitations of previous clinical trials to better investigate the effects of SGLT2 inhibitors on myocardial injury. However, it may have some limitations that should be acknowledged.

 The high variability of included studies and the limited number of clinical outcomes with different efficacy measures make conducting meta-analyses not applicable.

 The included studies in this systematic review have variability in types of administered SGLT2 inhibitors, evaluated cardiac injury biomarkers, and study populations. The investigated SGLT2 inhibitors were empagliflozin, dapagliflozin, and canagliflozin, each of which may have distinct pharmacodynamic properties influencing biomarkers of myocardial injury differently. Additionally, while all included studies assessed hs-cTn as the primary marker of myocardial injury, some studies focused on hs-cTnT while others evaluated hs-cTnI, which could contribute to differences in reported outcomes. The study populations also varied, with some trials including individuals with T2DM, while others focused on HF patients, potentially leading to different levels of baseline risk of cardiac injury and inflammation, resulting in different responses to SGLT2 inhibitors.

 Also, the majority of the included population was male, making it challenging to apply the findings to female individuals. Additionally, the majority of studies were conducted on White people. Hence, it may not be generalizable to other populations.

## Conclusion

 The outcomes of this systematic review may suggest an association between SGLT2 inhibitors and reduced hs-cTn levels in individuals with T2DM. However, the evidence in HF patients remains inconclusive, with most studies failing to demonstrate significant reductions in hs-cTn. Given the heterogeneity in study populations, types of cardiac injury biomarkers, and methodological rigor, further well-designed, large-scale randomized controlled trials are needed. Future research should stratify patients by disease phenotype (diabetes vs. heart failure), use standardized assays for cardiac injury biomarkers, and incorporate imaging modalities such as cardiovascular magnetic resonance (CMR) to quantify myocardial injury.

## Competing Interests

 The authors declare no conflicts of interest.

## Ethical Approval

 Not applicable.

## Supplementary Files

Supplementary file 1 contains Table S1.
