# Supplementary material for: SGLT2 Inhibitors’ Effects on Biomarkers of Myocardial Injury: A Systematic Review
Source: J Cardiovasc Thorac Res. 2025 Dec 20;18(2):74–81. doi: 10.34172/jcvtr.026.33445 (PMC13677609; doi:10.34172/jcvtr.026.33445)
Supplement: Supplementary file 1 [file jcvtr-18-74-s001.pdf]

J Cardiovasc Thorac Res 2026;18(2):74-S1
doi:10.34172/jcvtr.026.33445
https://jcvtr.tbzmed.ac.ir

Table S1. The detailed search strategy for investigated databases

Database	Search strategy		No. of results
	#Search No.	Query	
PubMed	#1	(("Cardiovascular Diseases"[Mesh] OR "Heart Diseases"[Mesh] OR angiocardiopathy OR “angiocardiovascular disease” OR “cardiovascular complication” OR “cardiovascular disease*” OR “cardiovascular disorder” OR “cardiovascular disturbance” OR “cardiovascular lesion” OR “cardiovascular syndrome” OR “cardiovascular vegetative disorder” OR complication, cardiovascular OR “disease, cardiovascular” OR “major adverse cardiovascular event” OR “cardiovascular disease” OR “cardiac anomaly” OR “cardiac disease” OR “cardiac disturbance” OR “cardiopathy” OR “heart deficiency” OR “heart deformity” OR “heart diseases” OR “heart disorder” OR “heart dysfunction” OR “heart disease” OR "Diabetes Mellitus"[Mesh] OR diabetes OR diabetic OR diabets OR “unspecified diabetes mellitus” OR “diabetes mellitus”))	10989511
	#2	(("Sodium-Glucose Transporter 2 Inhibitors"[Mesh] OR "Sodium-Glucose Transporter 2 Inhibitors" [Pharmacological Action] OR gliflozin* OR gliflozin derivative OR SGLT2 inhibitor OR SGLT2 inhibitors OR sodium dependent glucose cotransporter 2 inhibitor OR sodium glucose co-transporter 2	17778

	inhibitor OR sodium-glucose transporter 2 inhibitor* OR sodium glucose cotransporter 2 inhibitor OR canagliflozin OR atigliflozin OR bexagliflozin OR dapagliflozin OR empagliflozin OR enavogliflozin OR ertugliflozin OR ipragliflozin OR licogliflozin OR luseogliflozin OR mizagliflozin OR remogliflozin OR sergliflozin OR sotagliflozin OR tofogliflozin))	
#3	((“cardiac muscle lesion” OR “heart fiber injury” OR “heart muscle damage” OR “heart muscle lesion” OR “myocardial damage” OR “myocardial injury” OR “myocardial lesion” OR “myocardium damage” OR “myocardium injury” OR “myocardium lesion” OR “heart muscle injury” OR “cardiac damage” OR “cardiac injury” OR “cardiac lesion” OR “heart damage” OR “heart injuries” OR “heart lesion” OR “heart wound” OR injury, heart OR “heart injury” OR "Troponin"[Mesh] OR "Troponin T"[Mesh] OR "Troponin I"[Mesh] OR "Troponin C"[Mesh] OR troponin complex OR troponin component OR troponin* OR troponin C OR troponin I OR troponin T OR hs-ctni OR CPK MB OR CPK-MB OR cpk mb isoenzyme OR creatine kinase heart isoenzyme OR creatine kinase heart type OR creatine kinase isoenzyme 2 OR creatine kinase isoenzyme mb OR creatine kinase mb isoenzyme OR creatine kinase myocardial isoenzyme OR creatine kinase MB form OR creatine phosphokinase heart isoenzyme OR creatine phosphokinase heart type OR creatine phosphokinase mb OR CK-MB OR creatine phosphokinase mb isoenzyme OR MB creatine	275685

kinase OR myocardial creatine kinase OR creatine kinase MB OR H-FABP OR "FABP3 protein" OR "heart fatty acid binding protein" OR "heart-specific fatty acid binding protein" OR "heart-type fatty acid-binding protein" OR "mammary-derived growth inhibitor" OR "muscle fatty acid-binding protein" OR "protein FABP3" OR "fatty acid binding protein 3" OR "cardiac muscle myoglobin" OR "cardiac myoglobin" OR ferromyoglobin OR "heart muscle myoglobin" OR "heart myoglobin" OR "myocardial myoglobin" OR myoglobulin OR myohaemoglobin OR myohemoglobin OR myoglobin OR "(s) lactate:nad oxidoreductase" OR "e.c. 1.1.1.27" OR "l lactate dehydrogenase" OR "l lactate nad oxidoreductase" OR "l lactate nicotinamide adenine dinucleotide oxidoreductase" OR "l lactate:nad oxidoreductase" OR "l lactate:nicotinamide adenine dinucleotide oxidoreductase" OR "l lactic dehydrogenase" OR "l-lactate dehydrogenase" OR l.d.h. OR "lactate dehydrogenase*" OR lactatedehydrogenase OR "lactic acid dehydrogenase" OR "lactic dehydrogenase" OR lacticodehydrogenase OR lactodehydrogenase OR LDH OR "lactate dehydrogenase" OR "L-Lactate Dehydrogenase"[Mesh] OR “ischaemia modified albumin” OR “ischemia modified albumin” OR "ischemia-modified albumin" [Supplementary Concept] OR glycogen phosphorylase isoenzyme bb OR GPBB OR “glycogen phosphorylase BB” OR "glycogen phosphorylase BB, human" [Supplementary Concept]))

	#4	#1 AND #2 AND #3	483
Cochrane library	#1	("Cardiovascular Diseases" OR "Heart Diseases" OR angiocardiopathy OR “angiocardiovascular disease” OR “cardiovascular complication” OR “cardiovascular disease” OR “cardiovascular disorder” OR “cardiovascular disturbance” OR “cardiovascular lesion” OR “cardiovascular syndrome” OR “cardiovascular vegetative disorder” OR “complication, cardiovascular” OR “disease, cardiovascular” OR “major adverse cardiovascular event” OR “cardiovascular disease” OR “cardiac anomaly” OR “cardiac disease” OR “cardiac disturbance” OR “cardiopathy” OR “heart deficiency” OR “heart deformity” OR “heart diseases” OR “heart disorder” OR “heart dysfunction” OR “heart disease” OR "Diabetes Mellitus" OR diabetes OR diabetic OR diabets OR “unspecified diabetes mellitus”)	184517
	#2	((“Sodium-Glucose Transporter 2 Inhibitors” OR gliflozin* OR “gliflozin derivative” OR gliflozins OR “SGLT2 inhibitor” OR “SGLT2 inhibitors” OR “sodium dependent glucose cotransporter 2 inhibitor” OR “sodium glucose co-transporter 2 inhibitor” OR “sodium-glucose transporter 2 inhibitors” OR “sodium glucose cotransporter 2 inhibitor” OR canagliflozin OR atigliflozin OR bexagliflozin OR dapagliflozin OR empagliflozin OR enavogliflozin OR ertugliflozin OR ipragliflozin OR licogliflozin OR luseogliflozin OR mizagliflozin OR remogliflozin OR sergliflozin OR sotagliflozin OR tofogliflozin)) in All Text Keyword - (Word variations have been searched)	6530

#3	("cardiac muscle lesion" OR "heart fiber injury" OR "heart muscle damage" OR "heart muscle lesion" OR "myocardial damage" OR "myocardial injury" OR "myocardial lesion" OR "myocardium damage" OR "myocardium injury" OR "myocardium lesion" OR "heart muscle injury" OR "cardiac damage" OR "cardiac injury" OR "cardiac lesion" OR "heart damage" OR "heart injuries" OR "heart lesion" OR "heart wound" OR injury, heart OR "heart injury" OR "troponin complex" OR "troponin component" OR troponin* OR troponin C OR troponin I OR troponin T OR hs-ctni OR CPK MB OR CPK-MB OR cpk mb isoenzyme OR "creatine kinase heart isoenzyme" OR "creatine kinase heart type" OR "creatine kinase isoenzyme 2" OR "creatine kinase isoenzyme mb" OR "creatine kinase mb isoenzyme" OR "creatine kinase myocardial isoenzyme" OR "creatine kinase MB form" OR "creatine phosphokinase heart isoenzyme" OR "creatine phosphokinase heart type" OR "creatine phosphokinase mb" OR CK-MB OR "creatine phosphokinase mb isoenzyme" OR "MB creatine kinase" OR "myocardial creatine kinase" OR "creatine kinase MB" OR H-FABP OR "FABP3 protein" OR "heart fatty acid binding protein" OR "heart-specific fatty acid binding protein" OR "heart-type fatty acid-binding protein" OR "mammary-derived growth inhibitor" OR "muscle fatty acid-binding protein" OR "protein FABP3" OR "fatty acid binding protein 3" OR "cardiac muscle myoglobin" OR "cardiac myoglobin" OR	21386

		ferromyoglobin OR "heart muscle myoglobin" OR "heart myoglobin" OR "myocardial myoglobin" OR myoglobulin OR myohaemoglobin OR myohemoglobin OR myoglobin OR "(s) lactate:nad oxidoreductase" OR "e.c. 1.1.1.27" OR "l lactate dehydrogenase" OR "l lactate nad oxidoreductase" OR "l lactate nicotinamide adenine dinucleotide oxidoreductase" OR "l lactate:nad oxidoreductase" OR "l lactate:nicotinamide adenine dinucleotide oxidoreductase" OR "l lactic dehydrogenase" OR "l-lactate dehydrogenase" OR l.d.h. OR "lactate dehydrogenases" OR lactatedehydrogenase OR "lactic acid dehydrogenase" OR "lactic dehydrogenase" OR lacticodehydrogenase OR lactodehydrogenase OR LDH OR “lactate dehydrogenase” OR “ischaemia modified albumin” OR “ischemia modified albumin” OR glycogen phosphorylase isoenzyme bb OR glycogen phosphorylase BB OR GPBB) in All Text Keyword - (Word variations have been searched)	
	#4	#1 AND #2 AND #3	167
Embase	#1	'angiocardiopathy'/exp OR 'angiocardiopathy' OR 'angiocardiovascular disease'/exp OR 'angiocardiovascular disease' OR 'cardiovascular complication'/exp OR 'cardiovascular complication' OR 'cardiovascular diseases'/exp OR 'cardiovascular diseases' OR 'cardiovascular disorder'/exp OR 'cardiovascular disorder' OR 'cardiovascular disturbance'/exp OR 'cardiovascular disturbance' OR 'cardiovascular lesion'/exp OR 'cardiovascular lesion' OR 'cardiovascular syndrome'/exp OR	7243653

	'cardiovascular syndrome' OR 'cardiovascular vegetative disorder'/exp OR 'cardiovascular vegetative disorder' OR 'complication, cardiovascular'/exp OR 'complication, cardiovascular' OR 'disease, cardiovascular'/exp OR 'disease, cardiovascular' OR 'major adverse cardiovascular event'/exp OR 'major adverse cardiovascular event' OR 'cardiovascular disease'/exp OR 'cardiovascular disease' OR 'cardiac anomaly'/exp OR 'cardiac anomaly' OR 'cardiac disease'/exp OR 'cardiac disease' OR 'cardiac disturbance'/exp OR 'cardiac disturbance' OR 'cardiopathy'/exp OR 'cardiopathy' OR 'heart deficiency'/exp OR 'heart deficiency' OR 'heart deformity'/exp OR 'heart deformity' OR 'heart diseases'/exp OR 'heart diseases' OR 'heart disorder'/exp OR 'heart disorder' OR 'heart dysfunction'/exp OR 'heart dysfunction' OR 'heart disease'/exp OR 'heart disease' OR 'diabetes'/exp OR 'diabetes' OR 'diabetic'/exp OR 'diabetic' OR 'diabets'/exp OR 'diabets' OR 'unspecified diabetes mellitus'/exp OR 'unspecified diabetes mellitus' OR 'diabetes mellitus'/exp OR 'diabetes mellitus'	
#2	'gliflozin'/exp OR 'gliflozin' OR 'gliflozin derivative'/exp OR 'gliflozin derivative' OR 'gliflozins'/exp OR 'gliflozins' OR 'sglt2 inhibitor'/exp OR 'sglt2 inhibitor' OR 'sglt2 inhibitors'/exp OR 'sglt2 inhibitors' OR 'sodium dependent glucose cotransporter 2 inhibitor'/exp OR 'sodium dependent glucose cotransporter 2 inhibitor' OR 'sodium glucose co-transporter 2 inhibitor'/exp OR 'sodium glucose co-transporter 2 inhibitor' OR 'sodium-glucose	39261

	transporter 2 inhibitors'/exp OR 'sodium-glucose transporter 2 inhibitors' OR 'sodium glucose cotransporter 2 inhibitor'/exp OR 'sodium glucose cotransporter 2 inhibitor' OR 'atigliflozin'/exp OR atigliflozin OR 'bexagliflozin'/exp OR bexagliflozin OR 'canagliflozin'/exp OR canagliflozin OR 'dapagliflozin'/exp OR dapagliflozin OR 'empagliflozin'/exp OR empagliflozin OR 'enavogliflozin'/exp OR enavogliflozin OR 'ertugliflozin'/exp OR ertugliflozin OR 'ipragliflozin'/exp OR ipragliflozin OR 'licogliflozin'/exp OR licogliflozin OR 'luseogliflozin'/exp OR luseogliflozin OR 'mizagliflozin'/exp OR mizagliflozin OR 'remogliflozin'/exp OR remogliflozin OR 'sergliflozin'/exp OR sergliflozin OR 'sotagliflozin'/exp OR sotagliflozin OR 'tofogliflozin'/exp OR tofogliflozin	
#3	'cardiac muscle lesion'/exp OR 'cardiac muscle lesion' OR 'heart fiber injury'/exp OR 'heart fiber injury' OR 'heart muscle damage'/exp OR 'heart muscle damage' OR 'heart muscle lesion'/exp OR 'heart muscle lesion' OR 'myocardial damage'/exp OR 'myocardial damage' OR 'myocardial injury'/exp OR 'myocardial injury' OR 'myocardial lesion'/exp OR 'myocardial lesion' OR 'myocardium damage'/exp OR 'myocardium damage' OR 'myocardium injury'/exp OR 'myocardium injury' OR 'myocardium lesion'/exp OR 'myocardium lesion' OR 'heart muscle injury'/exp OR 'heart muscle injury' OR 'cardiac damage'/exp OR 'cardiac damage' OR 'cardiac injury'/exp OR 'cardiac injury' OR 'cardiac lesion'/exp OR 'cardiac lesion' OR	388797

	'heart damage'/exp OR 'heart damage' OR 'heart injuries'/exp OR 'heart injuries' OR 'heart lesion'/exp OR 'heart lesion' OR 'heart wound'/exp OR 'heart wound' OR 'injury, heart'/exp OR 'injury, heart' OR 'heart injury'/exp OR 'heart injury' OR 'troponin complex'/exp OR 'troponin complex' OR 'troponin component'/exp OR 'troponin component' OR 'troponin'/exp OR 'troponin' OR 'troponin c'/exp OR 'troponin c' OR (('troponin'/exp OR troponin) AND ('c'/exp OR c)) OR 'troponin i'/exp OR 'troponin i' OR (('troponin'/exp OR troponin) AND i) OR 'troponin t'/exp OR 'troponin t' OR (('troponin'/exp OR troponin) AND t) OR 'hs ctni' OR 'cpk mb'/exp OR 'cpk mb' OR 'cpk mb isoenzyme'/exp OR 'cpk mb isoenzyme' OR 'creatine kinase heart isoenzyme'/exp OR 'creatine kinase heart isoenzyme' OR 'creatine kinase heart type'/exp OR 'creatine kinase heart type' OR 'creatine kinase isoenzyme 2'/exp OR 'creatine kinase isoenzyme 2' OR 'creatine kinase isoenzyme mb'/exp OR 'creatine kinase isoenzyme mb' OR 'creatine kinase mb isoenzyme'/exp OR 'creatine kinase mb isoenzyme' OR 'creatine kinase myocardial isoenzyme'/exp OR 'creatine kinase myocardial isoenzyme' OR 'creatine kinase, mb form'/exp OR 'creatine kinase, mb form' OR 'creatine phosphokinase heart isoenzyme'/exp OR 'creatine phosphokinase heart isoenzyme' OR 'creatine phosphokinase heart type'/exp OR 'creatine phosphokinase heart type' OR 'creatine phosphokinase mb'/exp OR 'creatine phosphokinase mb' OR 'creatine phosphokinase mb isoenzyme'/exp OR 'creatine

	phosphokinase mb isoenzyme' OR 'mb creatine kinase'/exp OR 'mb creatine kinase' OR 'myocardial creatine kinase'/exp OR 'myocardial creatine kinase' OR 'creatine kinase mb'/exp OR 'creatine kinase mb' OR 'h fabp' OR 'fabp3 protein'/exp OR 'fabp3 protein' OR 'fatty acid-binding protein, heart'/exp OR 'fatty acid-binding protein, heart' OR 'heart fatty acid binding protein'/exp OR 'heart fatty acid binding protein' OR 'heart-specific fatty acid binding protein'/exp OR 'heart-specific fatty acid binding protein' OR 'heart-type fatty acid-binding protein'/exp OR 'heart-type fatty acid-binding protein' OR 'mammary-derived growth inhibitor'/exp OR 'mammary-derived growth inhibitor' OR 'muscle fatty acid-binding protein'/exp OR 'muscle fatty acid-binding protein' OR 'protein fabp3'/exp OR 'protein fabp3' OR 'fatty acid binding protein 3'/exp OR 'fatty acid binding protein 3' OR 'cardiac muscle myoglobin'/exp OR 'cardiac muscle myoglobin' OR 'cardiac myoglobin'/exp OR 'cardiac myoglobin' OR 'ferromyoglobin'/exp OR 'ferromyoglobin' OR 'heart muscle myoglobin'/exp OR 'heart muscle myoglobin' OR 'heart myoglobin'/exp OR 'heart myoglobin' OR 'myocardial myoglobin'/exp OR 'myocardial myoglobin' OR 'myoglobin, heart muscle'/exp OR 'myoglobin, heart muscle' OR 'myoglobulin'/exp OR 'myoglobulin' OR 'myohaemoglobin'/exp OR 'myohaemoglobin' OR 'myohemoglobin'/exp OR 'myohemoglobin' OR 'myoglobin'/exp OR 'myoglobin' OR '(s) lactate:nad oxidoreductase'/exp OR '(s) lactate:nad

oxidoreductase' OR 'e.c. 1.1.1.27'/exp OR 'e.c. 1.1.1.27' OR 'l lactate dehydrogenase'/exp OR 'l lactate dehydrogenase' OR 'l lactate nad oxidoreductase'/exp OR 'l lactate nad oxidoreductase' OR 'l lactate nicotinamide adenine dinucleotide oxidoreductase'/exp OR 'l lactate nicotinamide adenine dinucleotide oxidoreductase' OR 'l lactate:nad oxidoreductase'/exp OR 'l lactate:nad oxidoreductase' OR 'l lactate:nicotinamide adenine dinucleotide oxidoreductase'/exp OR 'l lactate:nicotinamide adenine dinucleotide oxidoreductase' OR 'l lactic dehydrogenase'/exp OR 'l lactic dehydrogenase' OR 'l-lactate dehydrogenase'/exp OR 'l-lactate dehydrogenase' OR 'l.d.h.'/exp OR 'l.d.h.' OR 'lactate dehydrogenases'/exp OR 'lactate dehydrogenases' OR 'lactatedehydrogenase'/exp OR 'lactatedehydrogenase' OR 'lactic acid dehydrogenase'/exp OR 'lactic acid dehydrogenase' OR 'lactic dehydrogenase'/exp OR 'lactic dehydrogenase' OR 'lacticodehydrogenase'/exp OR 'lacticodehydrogenase' OR 'lactodehydrogenase'/exp OR 'lactodehydrogenase' OR 'ldh'/exp OR 'ldh' OR 'lactate dehydrogenase'/exp OR 'lactate dehydrogenase' OR 'ischaemia modified albumin'/exp OR 'ischaemia modified albumin' OR 'ischemia modified albumin'/exp OR 'ischemia modified albumin' OR 'glycogen phosphorylase isoenzyme bb'/exp OR 'glycogen phosphorylase isoenzyme bb' OR (('glycogen'/exp OR glycogen) AND ('phosphorylase'/exp OR phosphorylase) AND ('isoenzyme'/exp OR isoenzyme) AND bb) OR 'glycogen

		phosphorylase bb'/exp OR 'glycogen phosphorylase bb' OR (('glycogen'/exp OR glycogen) AND ('phosphorylase'/exp OR phosphorylase) AND bb) OR gpbb	
	#3	#1 AND #2 AND #3	1120
